# Neural Correlates of Enhanced Visual Short-Term Memory for Angry Faces: An fMRI Study

**DOI:** 10.1371/journal.pone.0003536

**Published:** 2008-10-29

**Authors:** Margaret C. Jackson, Claudia Wolf, Stephen J. Johnston, Jane E. Raymond, David E. J. Linden

**Affiliations:** School of Psychology, Bangor University, Bangor, Gwynedd, United Kingdom; University of California Davis, United States of America

## Abstract

**Background:**

Fluid and effective social communication requires that both face identity and emotional expression information are encoded and maintained in visual short-term memory (VSTM) to enable a coherent, ongoing picture of the world and its players. This appears to be of particular evolutionary importance when confronted with potentially threatening displays of emotion - previous research has shown better VSTM for angry versus happy or neutral face identities.

**Methodology/Principal Findings:**

Using functional magnetic resonance imaging, here we investigated the neural correlates of this angry face benefit in VSTM. Participants were shown between one and four to-be-remembered angry, happy, or neutral faces, and after a short retention delay they stated whether a single probe face had been present or not in the previous display. All faces in any one display expressed the same emotion, and the task required memory for face identity. We find enhanced VSTM for angry face identities and describe the right hemisphere brain network underpinning this effect, which involves the globus pallidus, superior temporal sulcus, and frontal lobe. Increased activity in the globus pallidus was significantly correlated with the angry benefit in VSTM. Areas modulated by emotion were distinct from those modulated by memory load.

**Conclusions/Significance:**

Our results provide evidence for a key role of the basal ganglia as an interface between emotion and cognition, supported by a frontal, temporal, and occipital network.

## Introduction

Visual short-term memory (VSTM) is an active system that temporarily stores and updates information over a period of a few seconds. It is particularly useful for maintaining a constant and coherent percept of the world in the face of eye, head, and object motion. In contrast, long-term memory (LTM) is a system dedicated to storing information over hours, days, and even decades; it is essential for learning and developing knowledge and skills.

Although it is well established that LTM is enhanced for images with an emotional, particularly negative, content [1]–[3], an effect thought to be driven by neural communication between LTM and limbic systems [4], the question of whether information to be retained in VSTM is influenced by its emotional content, and which brain mechanisms might be involved, has received little attention and results are varied. One study found no effect of valence on STM for fearful versus neutral faces, nor for taboo versus neutral words [5]. Two studies using emotive images from the International Affective Picture System (IAPS) found an influence of valence on STM. In one, participants judged the relative emotional intensity (“higher” or “lower”) of two successively presented images that were matched for valence (positive or negative) and were separated by a 3 second retention interval [6]. Young participants were more likely to make accurate relativity judgments for negative compared to positive images (accuracy was based on whether judgments matched previously established ratings obtained from an independent group of young participants). The authors interpreted this to reflect enhanced STM for negative images, and report the opposite effect with older participants. However, their task was not a direct test of the effect of valence on STM for visual content per se. In a functional magnetic resonance imaging (fMRI) study, participants were required to state whether a positive, negative, or neutral image seen 11.5 seconds earlier was present or not in an array of nine valence-matched images [7]. Increased activity in dorsolateral prefrontal cortex (DLPFC) and decreased orbitofrontal cortex (OFC) activity was found for positive versus negative images, but these data are difficult to interpret because task accuracy during scanning did not show a difference in STM for positive (65%) versus negative (65%) images. The above studies, while interesting in measuring responses to emotional stimuli, provide little clear insight into whether visual information with an emotional content can influence VSTM and, if so, what brain mechanisms might be involved. [Note that because our aim is to measure the neural correlates of information retained in VSTM with an emotional versus neutral content, we do not review here studies of the effect of emotional distraction or induced mood state on VSTM for neutral stimuli.]

Previous behavioural research of ours [8] has shown that VSTM for face identities is significantly enhanced when faces display an angry compared to a happy or neutral expression. We replicated this result a number of times and were able to eliminate several possible accounts of the effect. We showed that that the anger benefit for faces in VSTM was not due to low-level feature recognition: inverting the faces abolished the effect; a perceptual discrimination task in which participants stated whether two faces matched identity or not showed no difference in accuracy or reaction time between angry, happy, or neutral face conditions. We also showed that heightened physiological arousal is unlikely to underpin the effect: the presence of calming or energizing background music during the task did not differentially influence VSTM nor interact with emotional expression conditions, suggesting that enhanced VSTM for angry faces is valence-driven. Perceptual encoding limitations were excluded as an account because the angry benefit remained present when the original study time of 2000 ms was doubled. Finally, the effect was replicated using a different set of faces from another database that were also equated for expression intensity, providing evidence that enhanced VSTM for angry faces is not specific to the faces used, nor due to the potential for angry faces to be more intense in expression than happy or neutral faces.

In the current study, we again used angry, happy, and neutral faces to investigate the neural correlates of VSTM for information with an emotional versus neutral content. Faces are well suited for this purpose because not only are they ecologically valid, they also allow the presentation of differently valenced emotional information in the same individual exemplars. This reduces variability of low-level featural information among different emotion conditions, a factor that may have confounded results of previous studies using IAPS pictures [9]. Another person's emotional facial expression can convey critical information about his/her internal mood state and, in turn, affect one's own behavioral decisions, e.g., whether to approach or avoid, or what manner of speech to adopt. Successful and appropriate face-to-face interactions depend not only on recognition of emotional expression, but often also require accurate face identification. Critically, our ability to select an appropriate social response in a timely and effective manner depends on our ability to identify who is expressing what emotion, and this information must be retained in memory for a period sufficient to develop an action plan. Thus, storage of face identity information in VSTM forms a crucial bridge between immediate encoding of emotionally charged information and execution of appropriate behavior.

**Figure 1:**
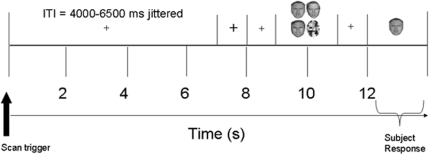
Here is an example trial procedure (load 3 shown as illustration). Between one and four faces (all expressing either angry, happy, or neutral emotion) were shown for encoding for 2000 ms, followed by a 1000 ms blank retention/maintenance phase, and a 2000 ms retrieval phase in which participants stated whether a single probe face had been present or not in the previous display. All faces in any one trial (i.e., at encoding and retrieval) displayed the same emotion. A jittered inter-trial interval (ITI) of between 4000 ms and 6500 ms separated each trial.

Here, during fMRI participants were required to memorize between one and four angry, happy, or neutral faces for 2,000 ms (the number of faces to be remembered is termed *face load*), and one second later they were asked to report whether a single face probe matched in identity to one of the previous to-be-remembered faces or not (Fig. 1). All faces (at both encoding and retrieval) in any one trial displayed the same emotion, thus emotional expression of the to-be-remembered faces was task-irrelevant. Our aim was to specifically examine the neural correlates of the angry benefit for faces in VSTM and determine how emotion and memory systems in the brain might interact to produce this effect. By manipulating face load, we were also able to examine any interactions between load and expression conditions. We predicted that the angry face benefit in VSTM is likely to recruit an interplay of brain regions involved in emotion processing, such as the amygdala, basal ganglia, and insula [9]–[11], short-term memory, such as the prefrontal cortex [12], and face processing, such as the fusiform gyrus [13] and superior temporal sulcus [14].

## Methods

### Participants

Thirty five right-handed healthy volunteers (mean age 29 years; 15females) from the student and community panels in Bangor participated in return for £20. Subjects reported no history of neurological or psychiatric disorder, had normal or corrected to normal vision, and provided informed written consent prior to participation. The study was approved by the School's ethics committee in Bangor.

### Stimuli

Greyscale face images of six adult males each expressing three emotions (angry, happy, and neutral) were used [15]. Each image subtended approximately 1.43°×1.36°. Scrambled greyscale face images, selected at random from a set of eight different scrambled images, were used to fill memory display locations on trials in which fewer than four faces were presented.

### Experimental Procedure

Participants were oriented to the centre of the computer screen by a small fixation cross presented for 1,000 ms and instructed to maintain fixation throughout each session in order to minimize eye movement artefacts in the functional data. To signal the start of a trial, the fixation cross increased in size for 1,000 ms, after which it returned to its original size for another 1,000 ms. On each trial, between one and four faces, each expressing the same emotion (angry, happy, or neutral) were presented for 2,000 ms in a 2×2 memory matrix with fixation at the centre. The centre of each image within the matrix was positioned at a visual angle of approximately 1.27° from fixation to ensure that the faces display was foveal, and thus minimize eye movements. Previous research has established that 2,000 ms is sufficient time to encode four faces [8], [16]. On trials in which fewer than four faces were presented, all other matrix locations were occupied by a scrambled face. Face locations were randomised within the matrix. After a 1,000 ms blank retention interval during which only the fixation cross was present, a single face probe (expressing the same emotion as the preceding matrix) was displayed in the centre of the screen for 2,000 ms. Participants were required to state, within the 2,000 ms single probe presentation duration, whether the probe person had been present or not in the immediately preceding display (50% probe present). The task involved an identity decision, thus emotional expression was irrelevant to the task. Participants used their right hand to respond “yes” or “no” using a simple button press. Feedback was not provided. A jittered fixation inter-trial interval (ITI) of between 4,000 and 6,500 ms separated each trial (Figure 1).

Sixteen experimental trials were presented for each load (1,2,3,4) in each emotion condition (angry, happy, neutral) in a pseudo-random order, resulting in 192 trials in total (event-related design). In order to minimize subject fatigue, the experiment was separated into four separate scanning blocks of 48 trials each, within a single scanning session. Each block lasted approximately 11 minutes. Before the main experiment began, participants were given a short practice session outside the scanner.

### Data Acquisition

Behavioural data were acquired with a 14-inch Dell Latitude D610 laptop (32-bit true colour; resolution 1280×1024 pixels). The tasks were generated by E-Prime software [17]. fMRI data were acquired with a Philips 1.5T MRI scanner with a SENSE parallel head coil. We used a gradient echo echoplanar sequence sensitive to the blood oxygen dependent (BOLD) signal (TR = 2,000 ms; TE = 40 ms; matrix size = 96×96; FOV = 256×256 mm^2^; voxel size = 3×3×3 mm^3^; 90° flip angle; 20 axial slices). Two dummy volumes were acquired before each scan block to reduce possible T1 saturation effects. During the VSTM faces task, the fMRI sequence was synchronized with the fixation cross at the start of each trial (see Fig. 1). Anatomical data was acquired with a high resolution T1-weighted three-dimensional (3D) volume (1×1×1 mm^3^), and used for coregistration of functional data.

### Data Analysis

#### Behavioural Data Analysis

False alarm rates in all emotional expression conditions varied significantly as a function of face load, so we converted hits and false alarms into dprime (d') scores in order to provide a more sensitive measure of signal detection. d' is the z-normalised hit rate (probability of ‘yes’ responses when the probe was present) minus the z-normalised false alarm rate (probability of ‘yes’ responses when the probe was absent) [d' = _z_Hit Rate – _z_False Alarm Rate].

#### FMRI Data Analysis

Functional data were preprocessed and analysed using the BrainVoyager 1.79 software. We applied slice scan time correction using sinc interpolation and ascending slice scanning order, 3D motion correction using trilinear interpolation, spatial smoothing (8 mm Gaussian kernel), and a temporal high pass filter (3 cycles per time course). Three-D anatomical scans were transformed into Talairach space [18], the parameters of which were applied to the coregistered functional data.

All but one subject completed all four VSTM task runs (one subject completed only three runs due to technical scanning problems), and runs that were unsuitable for analysis were excluded from analysis (two runs in each of two subjects revealed head movements greater than 5 mm). In total, 135 z-normalised volume time courses were entered into a whole brain, random effects analysis of covariance (ANCOVA). Motion-corrected covariates were included in the model in order to optimize the elimination of task-correlated motion artifacts and maximize sensitivity to true activations [19], and to reduce inter- and intra-subject variability [20]. Functional data from all phases of the VSTM task (excluding the ITI) were entered into the analysis model: no distinctions were made between encoding, maintenance, or retrieval phases. In all analyses, regions of activation were determined using the False Discovery Rate (FDR) significance threshold of <.05. To examine emotional expression effects, we computed a repeated-measures ANCOVA (three within-factor levels: angry, happy, neutral) to assess the main effect of emotion, and we also computed specific emotion contrasts (angry - neutral, angry - happy, happy - neutral). In each identified emotion cluster, we conducted random effects GLM region of interest (ROI) analyses to extract beta values that were subsequently applied to statistical comparisons between emotional expression conditions, and correlated with VSTM task performance values. VSTM load effects were examined by contrasting loads 4, 3, and 2 with load 1. A repeated-measures ANCOVA with emotion and load as within factors assessed whether an emotion by load interaction was present at the whole brain level.

#### Correlation with behavioural data

To examine whether there were any correlations between the magnitude of the angry face effect and brain activity levels, we used the mean behavioural dprime score across all face loads for each emotional expression condition to calculate difference scores for angry minus happy and angry minus neutral face contrasts (based on the angry face advantage observed in the behavioural results). These performance difference scores were correlated with related beta difference scores extracted from emotion-sensitive brain areas. To examine whether there were any correlations between STM capacity and brain activity levels, we calculated Cowan's K capacity estimates at each load [load*(hits – false alarms)] [21], averaged across emotion conditions, with related beta values extracted from load-sensitive brain areas. K and beta values were concatenated across all loads for this statistical comparison. Pearson's correlation coefficient (r^2^) was used in all cases.

## Results

### Behavioural Results

**Figure 2:**
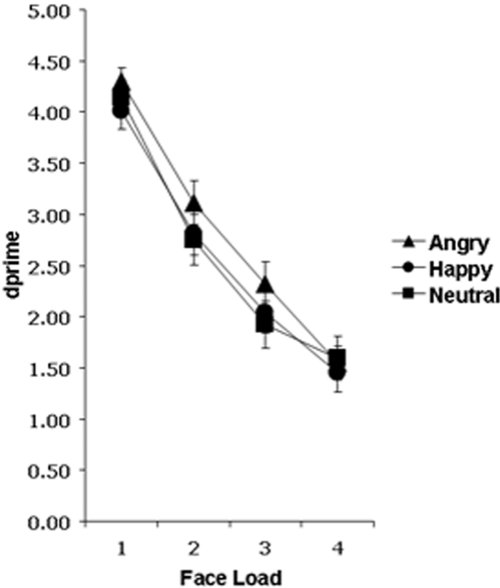
Behavioural performance on angry, happy, and neutral trials for all four face loads are displayed as d' (dprime) values. A maximum d' value of 4.66 indicates 100% performance, while a d' value of zero indicates performance at chance (50%). Participants performed significantly better on the VSTM task when the identities of angry faces were to be remembered, compared to happy or neutral faces. VSTM performance declined as face load increased for all emotional expression conditions. Bars represent±1 standard error.

We conducted an emotion (angry, happy, neutral) by load (1, 2, 3, 4) repeated-measures ANOVA on the behavioural data, expressed in d' values. Consistent with our previous findings [8], we found that VSTM performance was significantly modulated by emotional expression, *F*(2, 68) = 3.17, *p* = .048, and that angry faces were significantly better remembered than happy faces (*p*<.05) (Fig. 2). It is clear from Figure 2 that the effect of emotional expression appears most pronounced at face loads 2 and 3, likely due to the fact that we can only store about two face identities in VSTM at any one time [16]. When only face loads 2 and 3 are analysed, the main effect of emotion becomes more significant (*F*(2, 16) = 4.01, *p* = .02) and the difference between angry and neutral faces also reaches significance (*p*<.05). A significant main effect of face load was observed, *F*(3,102) = 120.38, *p*<.001, but its interaction with emotional expression was not significant, *F*(6, 204)<1.0.

### Functional Imaging Results

#### Emotion Effects

**Figure 3:**
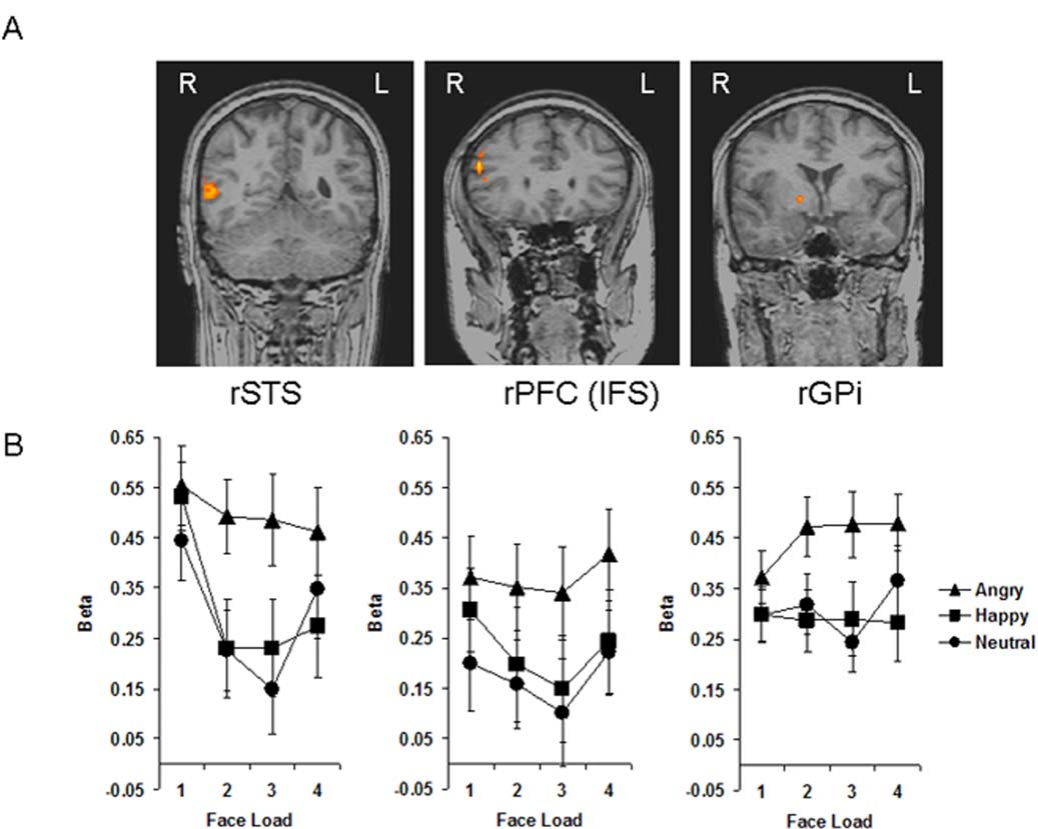
(A) Three coronal brain slices show modulation of brain activity by emotional expression of faces in the VSTM task in the superior temporal sulcus (STS), prefrontal cortex (PFC) along the inferior frontal sulcus (IFS), and globus pallidus internus (GPi), all in the right hemisphere. (B) Beta values for each emotion and face load condition are plotted for the STS, PFC, and GPi. Activity is greater for angry vs. happy and neutral face expression conditions in all three brain regions. Bars represent±1 standard error.

Using whole-brain analysis of variance (ANOVA), and an FDR significance threshold of *p*<.05, we found a significant main effect of emotion in three areas of the right hemisphere: superior temporal sulcus (STS), prefrontal cortex (PFC) along the anterior inferior frontal sulcus (IFS), and globus pallidus internus (GPi) (Fig. 3a). Talairach coordinates are provided in Table 1. There was no main effect of emotion in the left hemisphere. ROI analyses revealed that the main effect of emotion in the STS, PFC, and GPi was driven by significantly enhanced blood oxygen level dependent (BOLD) responses to angry faces (in all regions: angry vs. happy, *p*<.001; angry vs. neutral, *p*<.001) (Fig. 3b). There were no significant differences between happy and neutral face activations in any of these regions (*p*>.54 in all cases).

**Table 1:** Talairach coordinates and voxel cluster size values for the main effect of emotion (FDR<.05).

Region of activation	R/L	x	y	z	Cluster size (mm^3^)
STS	R	56	−52	7	1448
PFC (IFS)	R	54	29	20	320
GPi	R	16	−4	0	123

**Figure 4:**
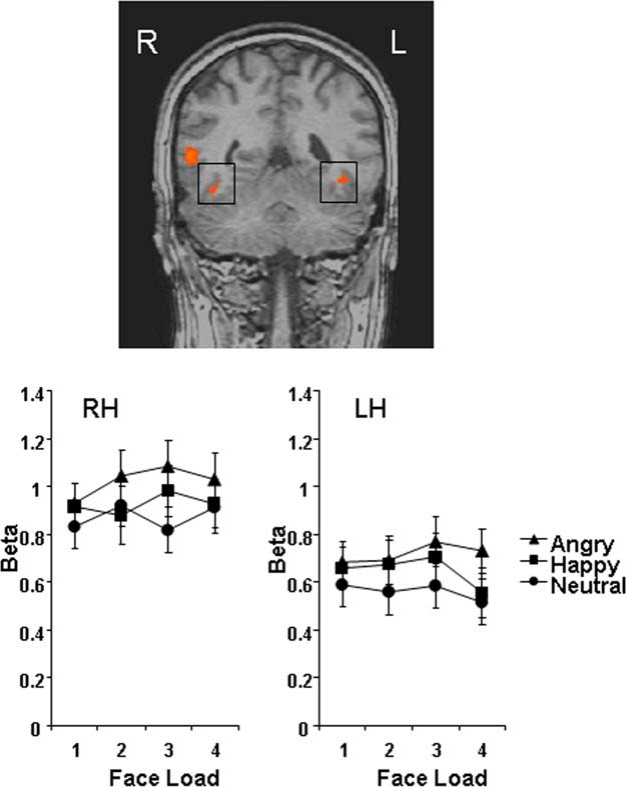
Coronal view shows bilateral fusiform activity obtained from the angry minus neutral contrast (regions outlined by black squares). Activity is greater for angry compared to neutral faces. Bars represent±1 standard error.

The angry minus neutral functional contrast showed the same pattern of activation as the main effect of emotion (higher activity for angry than neutral faces in rSTS, rPFC, and rGPi), but in addition this contrast revealed significantly greater angry vs. neutral activity in bilateral fusiform gyrus (*p*<.001 in both cases) (Fig. 4). In the right fusiform, analysis of extracted beta values also revealed significantly greater activation for angry vs. happy faces, *p* = .02. There were no load effects in these regions. At whole-brain level, the angry minus happy functional contrast similarly revealed rSTS activity (higher for angry) but did not show any additional regions of activation. No regions showed greater activation for happy vs. neutral faces. Talairach coordinates for the specific emotion contrasts are provided in Table 2.

**Table 2:** Talairach coordinates and voxel cluster size values for specific emotion contrasts (FDR<.05).

Contrast	Region of activation	R/L	x	y	z	Cluster size (mm^3^)
Angry-Neutral	STS	R	54	−54	6	2241
	PFC (IFS)	R	55	28	23	1353
	GPi	R	16	−2	3	16
	Fusiform	R	43	−43	−16	140
	Fusiform	L	−32	−42	−12	21
Angry-Happy	STS	R	57	−53	7	1282

The happy minus neutral contrast did not yield any regions of activation at this threshold.

#### Correlation Between Behavioral and Functional data for Emotion Effects

**Figure 5:**
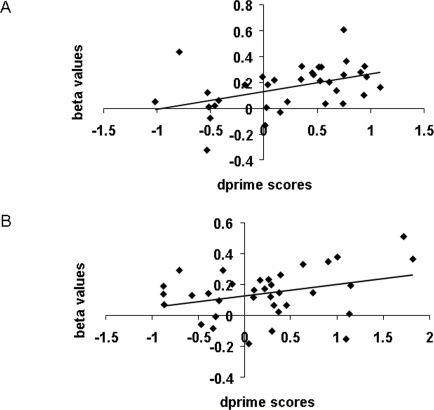
Better performance on the VSTM task for angry versus happy faces (A), and for angry versus neutral faces (B), was correlated with greater activity in the GPi.

To test whether higher activation for angry faces reflected a generalized increase in response to angry faces or associated arousal levels, or whether it might represent the very brain mechanism that brings about the angry face benefit in VSTM, we investigated the relationship between brain activity and behavioral data. We correlated the behavioral scores (difference in d') for the angry minus happy and angry minus neutral differences with the corresponding beta value differences in each emotion-sensitive region. In GPi, behavioural difference scores significantly correlated with related beta difference scores in the angry-happy contrast, *r* ^2^ = .44, *p* = .01 (Fig. 5a), and marginally correlated with related beta difference scores in the angry-neutral contrast, *r* ^2^ = .32, *p* = .06 (Fig. 5b). Superior VSTM for angry faces was thus correlated with enhanced activity in the GPi, suggesting a key role for this region in the angry face benefit. There were no significant correlations between behavioural scores and beta values in STS, PFC, or fusiform regions. Because the behavioural angry vs. neutral benefit was driven by the differences at loads 2 and 3, we re-ran these correlations using just loads 2 and 3. We replicated the angry-neutral contrast marginal correlation between behavioural and brain data in the GPi (*r* ^2^ = .33, *p* = .06), and additionally found a marginally significant angry-neutral contrast correlation in the right FFA (*r* ^2^ = .29, *p* = .09) suggesting perhaps some role of this face processing region in the angry vs. neutral benefit. Correlations in all other emotion-sensitive regions yielded a *p*-value greater than .10. We also correlated these behavioural data with related activity in load-sensitive areas and found no significant results.

#### Load Effects

**Figure 6:**
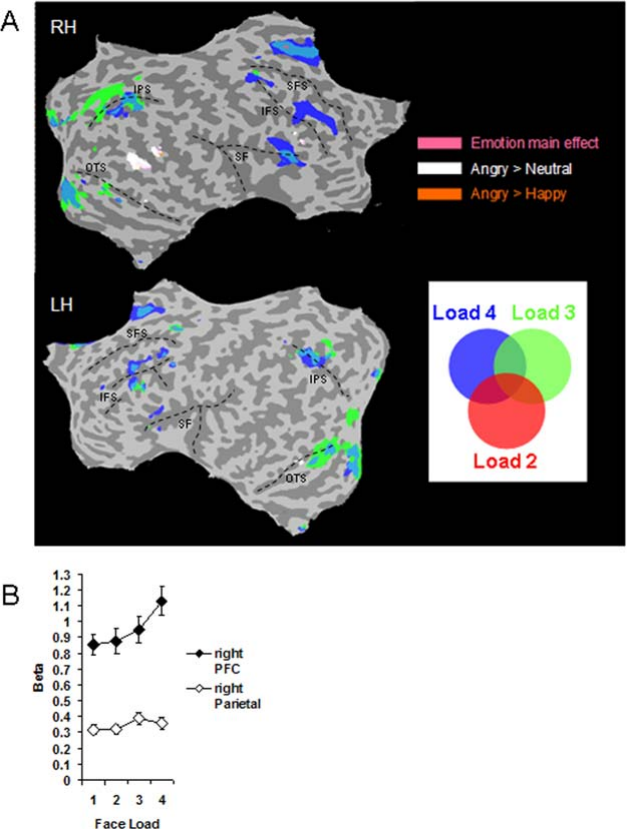
(A) Face loads 4 (blue), 3 (green), and 2 (red) were contrasted with face load 1. Several regions of the PFC, the frontal eye fields (FEF), inferior parietal sulcus (IPS), fusiform gyrus, and occipital cortex, in both left and right hemispheres, showed greater activity when multiple faces were to be remembered compared to just one face. Brain regions modulated by emotion in the right hemisphere (pink = emotion main effect; white = angry minus neutral contrast; brown = angry minus happy contrast) are overlain to illustrate the anatomical distinction between emotional expression and face load effects. Some anatomical landmarks are provided to aid navigation: superior frontal sulcus (SFS); inferior frontal sulcus (IFS); silvian fissure (SF); inferior parietal sulcus (IPS); occipito-temporal sulcus (OTS). (B) Beta values from each load condition (averaged across emotions) illustrate the contrast between a monotonic increase of activity with load in right PFC (x = 41, y = 29, z = 26) and peaked activation at load 3 in right parietal cortex (x = 18, y = −69, z = 43). Bars represent±1 standard error.

We examined load effects by contrasting loads 4, 3, and 2 with load 1, with the view that higher activity at loads greater than 1 indicates a greater draw on resources used to encode and retain multiple face identities in VSTM. Several areas in bilateral dorsolateral, ventrolateral, and medial prefrontal cortex (DLPFC, VLPFC, MPFC), frontal eye field (FEF), inferior parietal sulcus (IPS), fusiform gyrus, and occipital cortex showed significantly higher activation when multiple faces were to be remembered compared to one face in both the right and left hemispheres (Fig. 6a). These results conform to previous studies of face load in STM [22]. Interestingly, we replicated the dissociation of load effects between parietal and prefrontal areas described previously [23], with activity in parietal areas peaking at load 3 and prefrontal activity rising further towards load 4 in a monotonic fashion (Fig. 6b). This dissociation was supported by a significant load by region interaction between beta values in right parietal cortex and right PFC, *F*(3, 102) = 16.05, *p*<.001. Talairach coordinates for the load contrasts are provided in Table 3.

**Table 3:** Talairach coordinates and voxel cluster size values for face loads 4 minus 1, 3 minus 1, and 2 minus 1 contrasts (FDR<.05).

Contrast	Region of activation	R/L	x	y	z	Cluster size (mm^3^)
Load 4-1	Medial PFC		2	16	48	13929
	Dorso-ventral PFC	R	41	29	26	14874
	DLPFC	L	−43	7	43	10217
	VLPFC	L	−37	16	7	751
	Anterior frontal	L	−34	53	24	1732
	FEF	R	35	0	60	5028
	FEF	L	−43	6	44	10619
	IPS	R	29	−59	37	1583
	IPS	L	−25	−65	52	5806
	Fusiform	R	36	−66	−19	4533
	Fusiform	L	−36	−66	−19	6598
	Occipital cortex		7	−73	−10	24252
Load 3-1	Medial PFC		1	17	44	6937
	DLPFC	L	−50	16	31	2602
	VLPFC	R	34	21	8	1590
	FEF	R	30	−8	51	217
	FEF	L	−30	−10	58	1116
	IPS	R	18	−69	43	7477
	IPS	L	−21	−68	50	3162
	Fusiform	R	38	−67	−15	4903
	Fusiform	L	−30	−71	−12	10557
	Occipital cortex		−2	−75	−4	36825
Load 2-1	Medial PFC		1	11	51	441
	FEF	L	−30	−9	59	85
	Fusiform	L	−38	−67	−13	142

The spatial dissociation of emotion and face load effects on brain activation is particularly striking. Although both emotion and load effects were observed in parts of the right PFC, these areas did not anatomically overlap (Fig. 6a). Similarly, the load effect in bilateral fusiform gyrus was anatomically different to fusiform activity modulated by the angry minus neutral contrast (the emotion region lies more anterior to the load region). Furthermore, a whole brain statistical analysis did not reveal any areas that showed an interaction between emotion and load.

#### Correlation Between Behavioral and Functional data for Load Effects

We also examined correlations between STM capacity estimates, as indexed by Cowan's K, and brain activation levels in load-sensitive areas. K capacity estimates (collapsed across emotion conditions) were: load 1 = 0.93 (*SE* = .02*)*; load 2 = 1.48 (*SE* = .07); load 3 = 1.70 (*SE* = .11); load 4 = 1.71 (*SE* = .13). Significant or marginally significant positive correlations were found in all regions except left VLPFC and right fusiform (Table 4): as the number of faces stored in STM (K) increased, activity also increased. We correlated these K data with load activity in emotion-sensitive areas and found no significant results, confirming the spatial dissociation between emotion and load effects.

**Table 4:** Correlation between STM capacity estimates (K) and related beta values in load-sensitive regions. r^2^ values are provided with *p* values in brackets.

Region of activation	Correlation coefficient r^2^
Medial PFC	**.32 ** ***(<.001)***
r Dorso-ventral PFC	**.32 ** ***(<.001)***
l DLPFC	**.16 ** ***(.06)***
l VLPFC	.12 *(.17)*
Anterior frontal	**.18 ** ***(.04)***
r FEF	**.25 ** ***(<.01)***
l FEF	**.16 ** ***(.06)***
r IPS	**.21 ** ***(.01)***
l IPS	**.23 ** ***(<.01)***
r Fusiform	.14 *(.10)*
l Fusiform	**.27 ** ***(<.01)***
Occipital cortex	**.33 ** ***(<.001)***

## Discussion

Our behavioral results show that VSTM is significantly enhanced for face identities when faces display an angry compared to a happy or neutral expression, replicating previous findings [8]. It has been suggested that effects of emotion on memory require time to emerge, allowing effective consolidation of such memories [4]. Yet here, as in our previous study, we show that the effects of emotion on memory can be more immediate – emotional expression can influence visual short-term memory for faces.

In the present study, a network of emotion-sensitive areas comprised STS, PFC, and GPi, all in the right hemisphere, in keeping with the view that the right hemisphere is more involved in the processing and generation of emotions and affect than the left [24], [25]. The specific areas all fit into current models of emotion processing. The STS has been identified as a key area for the extraction of emotional information from faces [14], [26], [27] and more generally for the evaluation of others' intentions [28]. The STS has also been specifically implicated in processing various forms of anger [29]. Regions of the PFC have been implicated in experience [30] and observation [31] of negative mood, and higher activity in response to negative than positive images has been evidenced in regions of the right ventrolateral PFC specifically [32]. Integration of emotional state and STM processes in regions of bilateral PFC has also been reported [33]. The GPi, a subcortical structure, is a major part of the basal ganglia which, beyond their function in the extrapyramidal motor circuit, are involved in a variety of cognitive functions including emotion processing [34].

What is striking about the present findings is that the right STS, PFC, and GPi were specifically recruited in the service of VSTM for angry faces. The GPi seems to be the main region responsible for enhanced VSTM for angry faces, and this finding concurs with a recent study that showed a positive correlation between increased globus pallidus activity and increased STM capacity for simple objects [35]. This study also outlined the role of the globus pallidus as an attentional filter that allows only relevant information access to VSTM. It is possible in our study that enhanced GPi activity to angry faces in VSTM might reflect heightened attention to angry faces, driven by the saliency of potential threat. Threat (anger and fear) expressions have frequently been reported as especially good at capturing attention [36]–[38], even when task-irrelevant [39]. However, these studies involve the capture of attention of a single angry face in a display of differently valenced faces, while in our study all faces in any one VSTM trial displayed the same emotion, thus removing any such competition for attention between different expressions. Furthermore, there is also evidence of rapid attentional orienting to happy faces [40] and more generally to stimuli with high emotional relevance [41]. Perhaps attention was heightened in general during angry face trials, in order to facilitate encoding and maintenance of person identity information in VSTM in the context of potential threat.

The prominent role of the GPi, which was the key area where neural activity was significantly correlated with behavioural performance, is in keeping with recent findings on the role of dopamine in recognition of angry expression. Selective impairment of angry face perception has been linked to: lack of dopamine in Parkinson's disease, which affects the information processing capacity of the GP [42], [43]; treatment with antidopaminergic drugs [44]; and deep brain stimulation of the subthalamic nucleus [45], which is directly connected with the limbic part of the GP [10]. The present study shows that the GPi, one of the main relay stations of the basal ganglia, is not only responsive to emotional stimuli but aids their processing in a way that allows the effective handling of evolutionarily salient information.

A specific angry vs. neutral contrast also revealed a role for the fusiform gyrus - a face-selective area [46] - in the angry benefit, wherein BOLD activity was higher for angry than neutral faces bilaterally and for angry than happy faces in the right hemisphere. Modulation of activity in the fusiform region by facial expression has been reported previously during passive viewing, identity matching, and emotion recognition tasks. For example, there is evidence that fearful [47]–[49], happy [47], [50], and angry faces [13] elicit greater fusiform activity than neutral faces. However, our study is the first to report modulation of the fusiform gyrus by facial expression during a VSTM task.

Traditionally, the amygdala has been implicated in the processing of emotional stimuli and in the long-term retention of emotional events or images wherein activity is often suggested to reflect heightened physiological arousal, which is thought to mediate emotional learning via direct and indirect neural pathways subserving short and long-term memory [4]. In our study, however, we did not find significant influence of the amygdala on the enhancement of VSTM for angry faces. There are a couple of explanations for this. First, the amygdala does not respond selectively to negative emotion: studies have shown activation in response to images of happy and neutral faces [51]. Thus, it is possible that the emotion contrasts computed here did not reveal modulation of the amygdala if all three emotions recruited this region to the same degree. Second, the angry face effect in VSTM is likely driven by image valence (i.e., negativity) rather than physiological arousal (i.e., excitability). In our previous behavioural study [8] we showed that music-induced arousal states did not modulate VSTM performance in general nor interact with expression conditions. We also found that arousal ratings, as measured by the Self-Assessment Manikin (SAM) rating scale [52], did not differ between angry and happy faces. Our behavioural data thus make a general arousal account of enhanced VSTM for angry versus happy faces less likely.

With regard to load modulated brain regions, higher activity in the fusiform gyrus can be explained by the larger number of faces in the memory encoding display, and may also reflect the involvement of this area in VSTM processes [22]. The activation increase in parietal and prefrontal areas reflects their role in supporting the attentional, encoding, and storage requirements of higher memory loads [23], [53]. Importantly, the bilateral fusiform regions that displayed load effects (e.g., load 4 – load 1; LH: x = −36, y = −66, z = −19; RH: x = 36, y = −66, z = −19) were anatomically distinct from the more anterior fusiform regions that displayed an angry face benefit (LH: x = −32, y = −42, z = −12; RH: x = 43, y = −43, z = −16). Face processing regions in the occipito-temporal cortex have been segregated previously into two distinct regions, the fusiform face area (FFA) and the occipital face area (OFA), the former located more anterior to the latter [54]. Our Talairach coordinates for the emotion- and load-affected fusiform regions correspond nicely with reported right hemisphere FFA and OFA coordinates respectively (FFA: x = 39, y = −44, z = −18; OFA: x = 39, y = −64, z = −20). None of the other load-related areas showed an additional modulation of their activity by emotional expression. This suggests that the enhancement of VSTM capacity by the angry expression operates mainly through the recruitment of emotion and face processing networks rather than through recruitment of additional neurons in the classical fronto-parietal STM network. The positive correlation between capacity estimates (K) and brain activation levels in most load-sensitive regions in the occipital, temporal, parietal, and frontal cortices, reflecting increased activity as the number of stored faces increased, suggests that activity in both low-level perceptual and higher-level cognitive areas is modulated by the amount of facial information stored in STM.

We propose a new neural mechanism that supports the angry face benefit in VSTM by facilitating processing and extending memory capabilities. Studies have reported several areas of the fronto-parietal STM network that pose a bottleneck for memory storage at high loads because they cannot respond by further increasing their levels of activity [23], [53], [55]. Our study suggests that VSTM for faces is not only supported by the recruitment of areas that are modulated by load, but also by areas that respond categorically and automatically to the presence of a certain type of stimulus content, in this case, emotion. In the present study, enhanced VSTM capacity for angry faces would thus have been supported by communication between emotion-sensitive areas (STS, IFS, GPi, and FFA) and face identification and VSTM areas (PFC, IPS, OFA).

Our findings also provide further perspective to the debate on whether or not there is independence between face identification and emotional expression decoding processes. While some studies have indicated dissociable neural representations for identity processing in the fusiform gyrus and facial expression processing in the anterior STS [26], [27], others suggest that neural circuits underpinning identity and expression processes overlap [56], [57]. We show that, in VSTM at least, the impact of (angry) emotional expression on face identification tends not to be achieved by multi-functionality of one region but by communication between different process-specific regions responsive to face expression or load. The dissociation between anger and load effects in anterior (FFA) and posterior (OFA) regions of the fusiform gyrus respectively is a novel finding, and perhaps suggests a more complex, fine-grained functional organisation of this region in supporting both expression and face identification processes.

Finally, our discovery of the pivotal role of the GPi at the interface between emotion and cognition may have profound implications for clinical neuropsychiatry. Deficits of social cognition, such as extraction of meaning from facial expressions, may be core elements of the psychopathology of schizophrenia and mood disorders. Whether these are linked to changes in the basal ganglia will have to be explored in future research. The basal ganglia also are the main target of deep brain stimulation for movement disorders and increasingly also for behavioural disorders, and a better understanding of their non-motor functions would be of great clinical importance.
